# Development and Usability of a Text Messaging Program for Women With Gestational Diabetes: Mixed Methods Study

**DOI:** 10.2196/32815

**Published:** 2022-02-22

**Authors:** Rachel A Blair, Christine E Horn, Jennifer M Dias, Marie E McDonnell, Ellen W Seely

**Affiliations:** 1 Division of Endocrinology, Diabetes, and Hypertension, Department of Medicine Brigham and Women's Hospital Harvard Medical School Boston, MA United States

**Keywords:** gestational diabetes mellitus, SMS text messaging, mobile phone, mobile health, pregnancy, blood glucose self-monitoring

## Abstract

**Background:**

Gestational diabetes mellitus (GDM) affects 5%-10% of pregnancies and can lead to serious fetal and maternal complications. SMS text messaging is an effective way to improve diabetes management outside of pregnancy, but has not been well studied in GDM.

**Objective:**

This study aimed to perform user experience testing and assess usability and acceptability of an SMS text messaging program (Text 4 Success) for women with GDM.

**Methods:**

An automated 2-way texting program was developed. It included (1) reminders to check blood glucose levels, (2) positive feedback to user-reported glucose levels, (3) weekly educational messages, and (4) weekly motivational messages. For the user experience testing, women received simulated messages. For the usability study, women were enrolled in the program and received messages for 2 weeks. All women participated in semistructured interviews. For women in the usability study, data from glucose measuring devices were downloaded to assess adherence to self-monitoring of blood glucose (SMBG), measured as the percentage of recommended SMBG checks performed (a secondary outcome).

**Results:**

Ten women participated in user experience testing. Suggestions for optimization included further customization of message timing and minimization of jargon, which were incorporated. Ten women participated in the usability study. All 10 would recommend the program to other women with GDM. Participants liked the immediate feedback to glucose values. Suggestions included further flexibility of messages related to mealtimes and the ability to aggregate blood glucose data into a table or graph. Overall, adherence to SMBG testing was high at baseline (222/238 recommended checks, 93%). In comparing the week prior to the trial with the 2 weeks during the trial, there was a small but statistically insignificant difference (*P*=.48) in the percentage of recommended SMBG performed (median 93% [25th-75th IQR 89%-100%] vs median 97% [25th-75th IQR 92%-100%]).

**Conclusions:**

Overall, women with GDM would recommend the Text 4 Success in GDM program and think it is helpful for GDM self-management. The program was usable and acceptable. The program may be better suited to those who have low levels of adherence to SMBG at baseline or to women at time of their diagnosis of GDM. Adaptations to the program will be made based on user suggestions. Further study of SMS text messaging to improve SMBG in GDM is needed.

## Introduction

Gestational diabetes mellitus (GDM) is a common condition, affecting 5%-10% of pregnancies in the United States [1], and has important implications for maternal and child health. Poorly controlled GDM can cause adverse fetal outcomes including preterm delivery, neonatal hypoglycemia, and fetal demise, as well as increased maternal risk for preeclampsia, Cesarean sections, and other complications [2]. The cornerstone of management relies on lifestyle modification and self-monitoring of blood glucose (SMBG), typically 4 times daily. As soon as the diagnosis of GDM is made, women are asked to start intensive monitoring very quickly [2,3]. The blood glucose values obtained not only allow women to understand their blood glucose trends but are also essential for clinicians to tailor therapy, including if pharmacologic treatment is indicated and whether adjustments to dosing are needed. Without the crucial information from SMBG, women and their clinicians cannot work together for optimal glucose control during pregnancy. However, many women have difficulty adhering to this intensive monitoring [4-6], and a study found that women with poor adherence to SMBG are more likely to have poor pregnancy outcomes including preeclampsia [7]. Therefore, a mobile health intervention that could improve SMBG in GDM could be very impactful.

SMS text messaging programs have been shown to improve glycemic control in diabetes outside of pregnancy [8,9]. There is preliminary evidence that SMS text messaging programs are well-received by women with GDM [10,11], though more research is needed. Two-way texting is patient centered and may improve engagement in care but has not been well-studied in GDM. Additionally, the SMS text messaging technology can be applied remotely, which became very relevant during the COVID-19 pandemic [12].

SMS text messaging programs have the advantage of being highly accessible and easily scalable, compared with apps that are only available on smartphones and require downloading and opening for use, and are more expensive to develop [8,13,14]. For these reasons, we developed an automated 2-way SMS text messaging program designed to increase SMBG in women with GDM. This study aimed to first assess user experience (phase 1), followed by usability and acceptability of an SMS text messaging program for women with GDM (phase 2). As a secondary outcome, we aimed to gather data about the program’s effect on adherence to SMBG.

## Methods

### Development of the Text 4 Success in Gestational Diabetes Text Messaging Program

**Figure 1:**
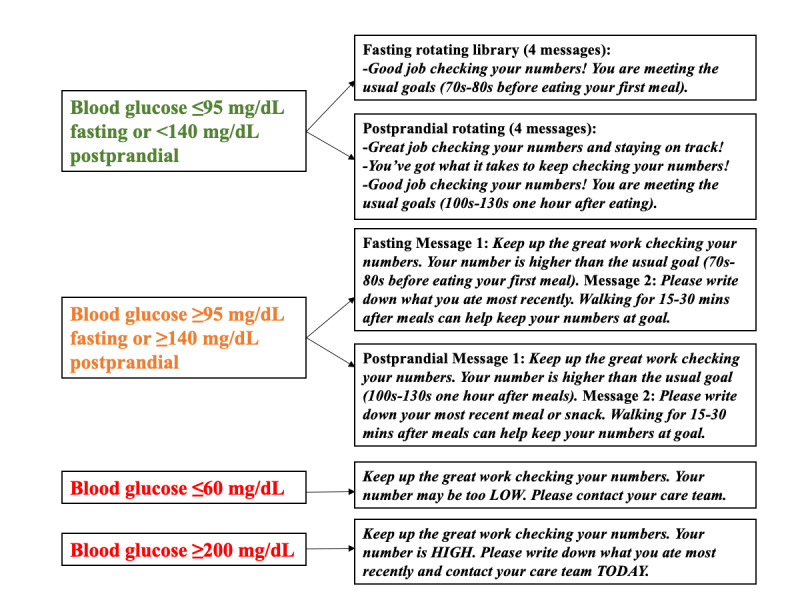
Algorithm for feedback on blood glucose.

We designed an automated SMS text messaging program for women with GDM called Text 4 Success in Gestational Diabetes (referred to as Text 4 Success). When designing the messages, we applied 2 components of the Health Belief Model: cue-to-action (the stimulus needed to take a health action) and self-efficacy (confidence in one’s ability to undertake a health action) [15]. The program included several different types of messages: (1) reminders to check blood glucose values fasting and 1 hour after meals (based on mealtimes reported at enrollment), (2) positive feedback on user-reported blood glucose values (with high or low values automatically prompting users to contact their clinical care team), (3) educational messages, and (4) motivational messages (Table 1). The reminders to check blood glucose values address the cue-to-action component of the Health Belief Model, providing an external stimulus to engage in SMBG [15]. The educational messages were designed to supplement information about GDM, physical activity, and healthy eating provided by clinicians. The positive feedback and motivational messages incorporate elements of self-efficacy from the Health Belief Model [15]. For instance, “You’ve got what it takes to keep checking your numbers!” specifically relates to a woman’s belief in her ability to carry out a behavior. More specific information about each component and sample messages are shown in Table 1 and Figure 1.

**Table 1:** Text 4 Success in Gestational Diabetes components.

Component	Description	Example
Reminders to check glucose values	Sent 4 times per day, 30 minutes before reported time of breakfast, and then 1 hour after reported time of breakfast, lunch, and dinner. Users were asked to text back their glucose values.	*Hello! This is a Text 4 Success reminder to check your number before eating your first meal. Please reply with your number only.*
Feedback on blood glucose values	In response to submitting glucose values, participants received encouraging feedback. In addition, an automatic algorithm sent messages based on glucose range (Figure 1).	*Keep up the great work checking your* *numbers* *and taking charge of your health!*
Educational messages	One per week sent at participants’ choice of time per day.	*Eating nutritious foods is a key part of staying healthy. One healthy snack is a plain Greek yogurt. Click for a list of more snacks:* *http://tinyurl.com/y2az4zn6*
Motivational messages	One per week sent at participants’ choice of time per day.	*If you feel off track, know that every day is another chance to get back on track!*

The messages were reviewed by a multidisciplinary group of experts, including endocrinologists, maternal–fetal medicine physicians, a behavioral psychologist, and a nutritionist. All information on the texting platform was at an eighth-grade reading level or below and available in English or Spanish based on participant preference. The program was specifically designed as an adjunct to clinical care. Therefore, participants were prompted by the text responses to contact their clinicians for both high and low glucose values that were out of desired range (fasting 61-94 mg/dL, postprandial 61-139 mg/dL). It was also designed in such a way that clinicians would not have the additional responsibility of monitoring the program in real time.

### Phase 1: User Experience Testing of the Text 4 Success Text Messaging Program

#### Participants

Women 18 years and older with GDM were recruited in Boston, Massachusetts, at a tertiary care center and affiliated clinics. Women were recruited by research assistants after clinical appointments as well as by clinicians seeing patients in maternal–fetal medicine obstetric clinics and endocrinology clinics focused on diabetes in pregnancy. A research assistant then obtained verbal informed consent. Participants received a parking voucher worth US $9 for participation.

At this institution, 2-step testing is used for the diagnosis of GDM [2]. GDM was defined by Carpenter–Coustan criteria applied to a 3-hour 100-g oral glucose tolerance test [16], a glucose value of ≥200 mg/dL after a 50-g glucose challenge test at >12 weeks of gestation, or a diagnosis of GDM documented in the electronic health record by a health care provider. Other inclusion criteria included completion of eighth grade, English or Spanish speaking, and ownership of a mobile phone with texting capability. Exclusion criteria included type 1 or type 2 diabetes or a hemoglobin A1c level ≥6.5% in the first trimester.

We performed user experience testing, defined as evaluation of a person’s responses as the result of the use of a system [17]. The goal of this process was to gather initial feedback on the design of the program as well as the phrasing and content of the messages in order to incorporate suggested changes.

#### Study Procedures

Members of our study team (RAB and JMD) met individually with each woman, sent simulated text messages from a study laptop to a study mobile phone held by the participant, and conducted semistructured interviews in English or Spanish to assess their opinions of the program. These meetings were in-person and took approximately 30-45 minutes to conduct. A research assistant (JMD) took notes during each interview. Messages were revised in an iterative manner based on user feedback. After the changes were made, we launched usability testing.

### Phase 2: Usability Testing

#### Participants

Inclusion criteria were similar to user experience testing. For usability testing, women additionally had to be ≤36 weeks of gestation and needed to have an unlimited SMS text messaging plan. Given that standard of care for GDM includes SMBG, all women were self-monitoring blood glucose typically using a glucometer or, in 1 case, a continuous glucose monitor. Women were recruited by clinicians at routine clinic visits. A research assistant then obtained verbal informed consent. Participants received a parking voucher worth US $22 for completing the study. They did not receive any specific compensation for replying to text messages.

#### Study Procedures

Women were enrolled in the SMS text messaging program for 2 weeks. The women started receiving text messages 24 hours after enrollment. Participants received a welcome message with opt-out information (users could easily unsubscribe by sending a text message stating “STOP”). They also received a message explaining the account is not monitored by a clinician in real time and to contact their care team with any clinical questions. They then answered a series of brief initial text messages with questions about mealtimes so that their reminders to check blood glucose could be timed accordingly. They were also sent baseline and end of study demographic questionnaires via a message on the secure patient portal used by the institution or by email, depending on patient preference. Questionnaires were designed and administered using REDCap (Research Electronic Data Capture) electronic data capture tools hosted at Harvard Catalyst, the Harvard Clinical and Translational Science Center [18,19]. REDCap is a secure, web-based software platform designed to support data capture for research studies. At the end of 2 weeks, women participated in semistructured interviews so we could obtain their feedback and assess their experience with the program. Interviews took approximately 30-45 minutes to complete and were conducted via Zoom, a cloud-based videoconferencing service offering features including online meetings and secure recording of sessions [20]. Audio-only calls were performed and the recording function was used to record the interviews.

After the study concluded, women were asked to drop off their glucometer or continuous glucose monitor at their clinic sites or mail their glucometer to the study staff using a prepaid shipping method. Data from these devices were then downloaded to determine the number of times that women were checking their blood glucose levels each day 1 week prior to and the 2 weeks during the study. We assessed the electronic health record to determine the number of blood glucose checks per day recommended by the clinician.

#### Measures and Analyses

Participants reported their age, race/ethnicity, and education level. We accessed the electronic medical records to determine gestational age at diagnosis of GDM as well as gestational age at enrollment into the study.

Likert scale questions were asked to assess usability and acceptability of the program as part of semistructured interviews performed at the end of 2 weeks. Participants were also asked open-ended questions about usability and acceptability of the program to better understand the Likert scale answers, as well as to obtain suggestions for improvement. This method of combining quantitative (Likert scale) and qualitative (open-ended) data has been used in a previous study [21]. Interview recordings were transcribed by an outside transcription service (Landmark Associates, Inc.). Two independent researchers (CEH and RAB) coded responses to determine themes, which were then organized by program component or as general program suggestions. Dedoose (Dedoose, LLC), a qualitative data program, was used to code the interview responses.

As a secondary outcome, we assessed adherence to recommended SMBG for the 1 week prior to enrollment compared with the 2 weeks during enrollment based on the data downloaded from glucose monitoring devices. The percentage of recommended glucose checks was determined by the total number of glucose levels checked divided by the total number of glucose checks recommended by the participant’s clinician. If a participant checked more often than the recommended checks, this percentage was still calculated as 100% of recommended checks.

### Statistical Analyses

The adherence rates for each participant were calculated for the week before and 2 weeks during the study. Adherence rates were assessed using the Shapiro–Wilk test for normality. To compare the SMBG adherence the week before and in the 2 weeks during the study, the Wilcoxon rank sum test was used. *P* values of <.05 were considered significant. Median and 25th-75th IQRs are reported.

### Ethics Approval

This study was approved by the Partners Healthcare Institutional Review Board, with the protocol numbers 2019P000010 for phase 1 and 2019P002591 for phase 2.

## Results

### Phase 1: User Experience Testing Results

We performed user experience testing with 10 women with GDM. Women were an average of 33.2 (3.2) years old. Five were White and non-Hispanic Latina, 3 were White and Hispanic/Latina, 1 was Black, and 1 was Asian. Four spoke English as their native language (3 spoke Spanish; 3 spoke other languages [Vietnamese, Haitian Creole, and Greek]). Two user experience sessions were conducted in Spanish using Spanish text messages and interview questions. Seven women had completed a college degree. Three were nulliparous and 3 had GDM in a prior pregnancy. Five were using insulin as treatment for GDM and 5 were on dietary therapy alone.

After the program was explained, all women expressed interest in using the SMS text messaging platform if it were available. Overall, women preferred messages without medical jargon. For example, our original welcome message was “Welcome to Text 4 Success in Gestational Diabetes (Txt4GDM)! You will receive reminders & info about GDM.” An early participant asked what the “M” stood for because GDM is not often referred to as “gestational diabetes mellitus” in conversations with patients. We therefore shortened the name of the program to Text 4 Success. Women suggested customization options, such as the option to pick the time of day to receive educational or motivational messages, which we incorporated. Finally, women wanted the messages to be more specifically related to GDM. For example, an educational message that said “Drinking water, instead of soda or juice, is healthy for you” was edited to include “and can help regulate your numbers” based on participant feedback.

### Phase 2: Usability Testing Results

#### Overview

Ten women underwent usability testing and their characteristics are shown in Table 2. The majority of participants were White and all were college educated. All reported receiving text messages from their doctors’ offices or pharmacies. No women replied “STOP” to unsubscribe to the text messages throughout the 2-week study period. On average, women were diagnosed 3 weeks prior to enrollment to the study, during which time they had been performing SMBG. Overall, women responded to 67.9% (380/560) of text messages that they received from the SMS text messaging program.

**Table 2:** Characteristics of the usability study population (n=10).

Characteristic			Value
Age (years), mean (SD)			36.5 (4.0)
Weeks of gestation at GDM^a^ diagnosis, mean (SD)^b^			27.3 (1.1)
Weeks of gestation at study enrollment, mean (SD)			30.4 (5.2)
**Race/ethnicity, n (%)**			
	White	9 (90)	
	Asian	1 (10)	
	Hispanic	0 (0)	
	Black	0 (0)	
	Multiple	0 (0)	
	Other	0 (0)	
**Native language, n (%)**			
	English	7 (70)	
	Other^c^	3 (30)	
**Highest reached education, n (%)**			
	Some or all of high school	0 (0)	
	Some college	0 (0)	
	College graduate	8 (80)	
	Graduate degree or higher	2 (20)	
Nulliparous, n (%)			3 (30)
GDM in prior pregnancy, n (%)			3 (30)
Used insulin during this pregnancy, n (%)			6 (60)

^a^GDM: gestational diabetes mellitus.

^b^Two participants had a clinician diagnosis of GDM and started monitoring blood glucose levels (using fingersticks) early in their pregnancy so we did not have a formal date of diagnosis for them. This value is the average for the other 8 participants.

^c^Other includes Farsi, Gujarati, and Hebrew.

#### Interview Results

All 10 participants completed the usability interview at the completion of the 2 week study. All participants stated they would recommend the program to other women with gestational diabetes. One participant mentioned that the program may be more helpful for women who do not check their blood glucoses frequently. Four women wanted to use the program for 2 weeks, 1 woman wanted to use it for 1-2 months, 4 wanted to use the program for their entire pregnancy, and 1 wanted flexibility to decide. Eight out of the 10 women thought the amount of text messages was just right (not too many or too few) and 2 thought there were too many messages. Seven of the 10 women said they would prefer an app, either for more flexible timing of reminders or for a way to aggregate blood glucose levels into a graph or table. One participant was neutral and 2 preferred SMS text messaging.

Responses to the Likert scale questions from all women assessing if the components of the SMS text messaging program were understandable and useful are shown in Table 3, using a Likert scale with 5 as the easiest or most helpful and 1 as not easy/not helpful. For helpfulness of reminders to check blood glucose levels, the mean was 3.3 (moderately helpful), with 5 women rating them as a 4 or 5, 2 women rating them as a 3, and 3 women rating them as a 1 or 2. Participants also reported aspects that they felt could be improved for future iterations. Below we describe the feedback for each component of the SMS text messaging program as well as overall suggestions for the program.

**Table 3:** User feedback on SMS text messaging components by Likert scale.

Component		Mean (SD)
**Four times daily reminders to check glucose**		
	Understandability^a^	5.0 (0.0)
	Helpfulness^b^	3.3 (1.3)
**Feedback messages to glucose values**		
	Understandability	4.8 (0.4)
	Helpfulness	3.7 (1.1)
**Educational texts**		
	Understandability	5.0 (0.0)
	Helpfulness	3.8 (0.9)
**Motivational texts**		
	Understandability	5.0 (0.0)
	Helpfulness	4.0 (1.3)

^a^Participants were asked for each type of message about understandability: on a scale from 1 to 5, where 1 is not easy, 3 is moderately easy, and 5 is very easy, how easy was it for you to understand these messages?

^b^Participants were asked for each type of message about helpfulness: On a scale from 1 to 5, where 1 is not helpful, 3 is moderately helpful, and 5 is very helpful, how helpful were these messages?

#### Reminders to Check Glucose Levels

Participants felt that reminders to check glucose levels, which included a request to text back in a glucose value, were easy to understand and helpful (Table 3). Participants liked that the reminders were brief and clear. One woman spoke specifically about how challenging it can be to suddenly have to engage in intensive monitoring and how the reminders were helpful in making that transition.

I think it can be overwhelming to start having to check from going to not doing this at all and then having to do it four plus times per day. Particularly for people who are really busy, it’s just so easy to forget. Even since I stopped getting the text messages, today I remembered it’d been two and a half hours since I last took my blood sugar. I think just getting those reminders made it a lot easier to remember to check.

Participants’ mealtimes varied day to day so they would have preferred more flexibility in being able to text in numbers at other times of the day rather than the set mealtimes entered at the beginning of the program.

It would almost work better if I could text in and say, “Just finished eating,”...and then it would give you a reminder that an hour later to test.

Two women suggested that if they did not reply to a reminder with their glucose value, a second reminder be sent after a given period.

#### Feedback to Glucose Values

Participants liked that the program had 2-way SMS text messaging and that feedback messages were sent back to them based on glucose values sent to the program. In addition, 2 women used the SMS text messaging chain as a way to track their glucoses, either transcribing them to tracking sheets or as a primary way to report them to their providers.

It was very nice that as soon as you input, it immediately tells you whether you’re in range or not. That’s super nice. If you don’t know what you actually have to be it will tell you you’re doing good or no, you should be in this range.

Four women found the text of the replies to glucose values repetitive and suggested more variety in the feedback responses. There were 4 rotating replies available when glucoses were in normal range, and only 1 reply if low (<60 mg/dL) or high (≥200 mg/dL).

I think having a wider range of responses that you get would be great. Some of the responses tended to be the same each day.

#### Educational Messages

Women appreciated practical educational tips, especially the text message that had a link to healthy snacks. Overall, 7 women wanted more than once weekly messages with educational content. Three suggested that the program could include more texts with links to additional information, such as exercise videos, healthy dessert recipes, or advice specifically around high fasting blood glucose.

Having only two messages in that week was like, okay, what else is there to know? I feel like there’s gotta be more, right? Especially maybe even frontloading more information early in the program would be nice.

#### Motivational Messages

Seven women found these messages quite encouraging and liked them.

When you get a discouraging number, it’s easy to get stuck in your head, so to just get a positive reinforcement like, “It's just one number. You can get back on track,” it was encouraging.

One woman did not find the motivational messages particularly helpful and 2 women were neutral. One woman suggested that the frequency of motivational messages could be adjusted based on user preferences, which could allow for women to select these types of messages more or less often depending on how helpful they found them.

#### General Program Suggestions

Overall, suggestions fell into 2 major categories: increased functionality and increased customizability. In terms of increased functionality, 7 participants suggested that there be some sort of graph or table that could aggregate their responses to the reminders.

It would be really nice if it was something that would generate a weekly report or something about your numbers since it’s taking that information that then you could share with the doctor or maybe could be automatically shared with your physician. I think something like that would be really, really helpful in, again, accountability and having a way to know when you’re overall on track or off track.

This type of graph or table functionality could either be achieved using an accompanying website associated with the texting platform or via an app. Specifically related to educational content, 1 woman suggested an app as that could have a more comprehensive library of information.

Second, women reported wanting increased customizability of the program. For example, 2 women specifically mentioned that they would have preferred to pick different mealtimes on weekdays compared with weekends. Two suggested that the educational messages could be tailored to areas of GDM management they were most struggling with, such as receiving educational messages specifically about managing fasting glucose levels.

#### Self-Monitoring of Blood Glucose Adherence Results

All participants had their glucose monitoring data downloaded from their glucose measuring devices (9 glucometers and 1 continuous glucose monitor).

We assessed the adherence to SMBG by calculating the percentage of recommended checks. The data for 1 week prior to the study were available for 9 out of 10 women because 1 woman was diagnosed and started SMBG at time of enrollment. Women were checking a median of 93% (25th-75th IQR 89%-100%) of recommended fingerstick blood glucose levels in the week prior to enrollment. During the 2 weeks in the trial, women checked a median of 97% (25th-75th IQR 92%-100%) of recommended glucose levels. The percentage of recommended fingerstick blood glucose levels was not significantly different when comparing the 1 week prior with the 2 weeks during the study (*P*=.48).

## Discussion

### Principal Findings

Overall, the Text 4 Success in Gestational Diabetes program was found to be usable and acceptable to women with GDM, with all women in the usability study saying they would recommend the program to other women with GDM. We used 2 components of the Health Belief Model to design the program: cue-to-action and self-efficacy [15]. The cue-to-action reminders were helpful according to the women in the study. The motivational messages targeting self-efficacy were well-received. The first step of user experience testing allowed us to refine messages and program structure prior to the usability study in an iterative fashion. In the 2-week usability study, 8 of 10 women thought the number of text messages (up to 4 reminders and 4 feedback messages per day) was just right. This number of text messages is higher than is usually seen for SMS text messaging programs during pregnancy (3-7 messages per week) [10,11,22]. There was a range in how long women wanted to use the program, with 4 reporting they would want to use it for their entire pregnancy.

Women reported that the program helped them make the rapid transition to SMBG 4 times daily, which is typically required in GDM. With high acceptability of the program, women were interested in further adaptations to the program, such as more flexible mealtimes, the ability to aggregate blood glucose values into a chart or graph, and more educational content. Although many stated they would prefer an app, some of the reasons they wanted an app could be addressed using a modified text message program alone or with an accompanying website. For example, for more flexible mealtimes, we could follow the suggestion of having users text in a keyword or keywords such as “ate meal” and then users would get a text reminder 1 hour later to check their blood glucoses. Additionally, an SMS text messaging program could have an accompanying website with a graph or table of glucose values and could include additional information about GDM as well. In addition to patients being able to see their blood glucose levels, clinicians could also view the data via a secure portal.

Alternatively, an app could be developed with push notifications for reminders to check blood glucose levels, more educational content, and summaries of glucose data. In fact, several apps for GDM have been developed without the specific focus of SMBG adherence. These apps have a range of features including graphs of glucose data, though few include specific reminders to check blood glucose levels [23,24]. When considering a future iteration of this program, it is notable that the cost of developing and maintaining an app is much more resource intensive than developing and maintaining an SMS text messaging program [14], and is thus more expensive [25,26]. Additionally, in resource-poor settings, fewer patients have access to smartphones with the ability to use apps [27]. A median of 76% of people in advanced economies own a smartphone and a median of 45% of people in emerging economies own a smartphone, whereas 94% of people in advanced economies and 83% of people in emerging economies own a mobile phone [27].

The study was not powered to detect differences in SMBG adherence comparing baseline with study duration and the intervention did not significantly increase the adherence rate for SMBG. Many women in the usability study had a very high adherence rate to SMBG (a median of 93%) prior to the study, which is higher than the average of approximately 70% adherence rate that has been described [28]. This high baseline adherence rate suggests that in the future, the program would be better suited to patients who have a lower baseline adherence to SMBG. The participants in the usability study all had a high level of education (college degree or higher) which may have played a role in their high baseline adherence rate [7].

### Limitations

There were several limitations to our study. The study was conducted at a single academic center. Participants were recruited by clinicians, so we do not have characteristics of those who declined participation. Participants in the usability testing all had a college degree or higher which could make findings less generalizable to patients of a lower socioeconomic status. The messages were well received in the user experience testing group as well, which had 3 participants without a college degree. The usability study only included 10 women, though other usability studies can have similar sample sizes [29,30]. The usability study did not include Spanish-speaking women. The usability testing only lasted 2 weeks so we do not have opinions on how the intervention would be received for a longer time frame. Participants only received 2 educational and 2 motivational text messages in the 2-week study, so feedback on those components is limited. The usability testing itself was not iterative, though it built upon feedback from the user experience testing. Finally, there was approximately a 3-week period between diagnosis of GDM and enrollment into the study, which may have led to the high baseline rates of glucose monitoring.

### Comparison With Prior Work

There have been a few recent studies assessing the impact of automated messaging on adherence to SMBG [11,31]. Johnson et al [11] conducted a 4-week intervention with 1-way texting in 19 women with GDM in the United States. One text message per day was sent, either a reminder to check blood glucose or an educational message. Nearly 67% felt the messages helped them remember to check glucose levels. In contrast to Johnson et al [11], our program gave immediate feedback to blood glucose levels via an algorithm, which women in our study reported that they found helpful.

In contrast to using an SMS text messaging program, several studies have used an app to increase SMBG. Peleg et al [31] designed an app that sent messages to user smartphones to encourage monitoring of a variety of different parameters including blood glucose in 19 participants with GDM in Spain. Glucometers were connected to user smartphones via Bluetooth and 4 reminders to check blood glucose levels were sent daily based on entered mealtimes. The system sent a message in response to elevated blood glucose readings to the patient and to the care provider. There was an improvement in mean adherence to SMBG in the intervention group (101%, SD 10%) compared with mean adherence in a historical control of 247 patients (87%, SD 28%; *P*=.03). Adherence was calculated such that it could be >100%. Neither Johnson et al [11] nor Peleg et al [31] described basing their program on an underlying health behavior change theory and neither mentioned soliciting patient input in the message development process.

There have been 3 randomized controlled trials of interventions conducted in Israel, China, and the United Kingdom that involved frequent communication with clinicians facilitated by an app and evaluated the effect on compliance to glucose monitoring [28,32,33]. Data on glucose levels were transmitted via Bluetooth from glucometers (or could be manually entered into the app in 1 study [32]). These 3 studies were similar to one another in that all required intensive communication from clinicians to participants (either daily or 3 times per week). None of the studies mentioned how these responsibilities were balanced with other clinical care. All showed improvement not only in adherence to SMBG but also in glycemic control.

There was one recent study examining a 1-way SMS text messaging program in GDM that was not specifically related to SMBG adherence [10]. The program sent 3 supportive or educational messages per week. Participants felt that the messages helped their motivation for diabetes self-care. Similar to our findings, participants wanted more educational and supportive messages and also desired more recipes.

### Conclusions

Our program is a novel 2-way texting program designed for women with GDM consisting of automated reminders and feedback to patients about their blood glucose values without requiring clinical staff to manage messages in real time. It allows feedback to be given by an algorithm rather than using clinician time, which has been brought up as a criticism of 2-way texting scalability [8]. Two components of the Health Belief Model (cue-to-action and self-efficacy), along with patient input, were used to design and refine the program. The program was easily understood and well received. The program may be better suited to women with a low baseline adherence rate to SMBG or to women at the time of their diagnosis of GDM. Women provided suggestions to improve the program, including having more customizability and functionality, which could be achieved with an accompanying website or by conversion to an app. These suggestions will be incorporated into the next iteration of the intervention. Further study, including a randomized controlled trial, is needed to assess this SMS text messaging program on adherence to SMBG.

## Abbreviations

GDM: gestational diabetes mellitus
SMBG: self-monitoring of blood glucose
